# Supplementary material for: Comparison of the Cox Proportional Hazards Model and Random Survival Forest Algorithm for Predicting Patient‐Specific Survival Probabilities in Clinical Trial Data
Source: Biom J. 2026 Sep 25;68(5):e70171. doi: 10.1002/bimj.70171 (PMC13615326; doi:10.1002/bimj.70171)
Supplement: Supplementary file 2 [file BIMJ-68-e70171-s002.zip › 2_local_analysis/MayoClinic/figures/cens_06/FigC3b_Mayo_06_ibs_beta_08.pdf]

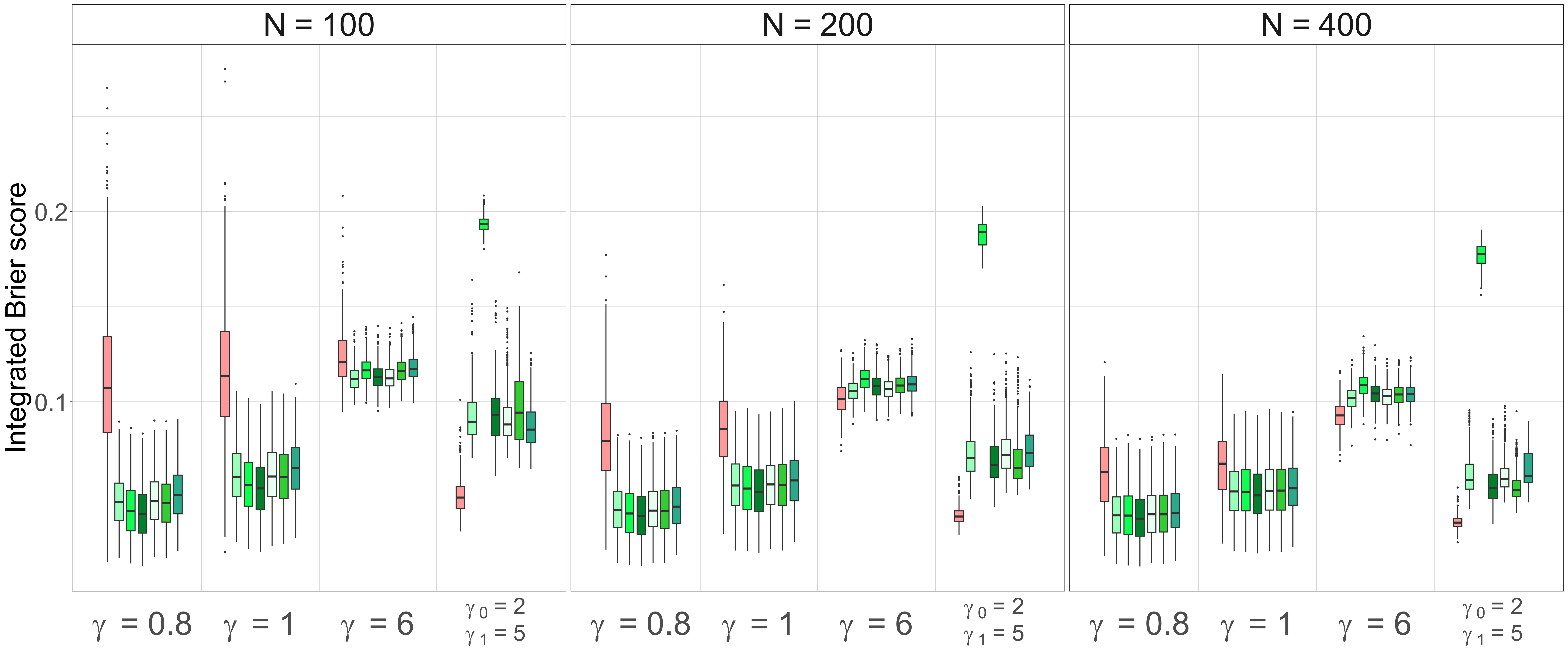
N = 100
N = 200
N = 400
Integrated Brier score
0.2
0.1
γ = 0.8
γ = 1
γ = 6
γ0 = 2
γ1 = 5
